# Characterization of the virulence of *Pseudomonas aeruginosa* strains causing ventilator-associated pneumonia

**DOI:** 10.1186/s12879-020-05534-1

**Published:** 2020-12-01

**Authors:** Beatriz Alonso, Laia Fernández-Barat, Enea Gino Di Domenico, Mercedes Marín, Emilia Cercenado, Irene Merino, Manuela de Pablos, Patricia Muñoz, María Guembe

**Affiliations:** 1 Department of Clinical Microbiology and Infectious Diseases, Hospital General Universitario Gregorio Marañón, Madrid, Spain; 2 Instituto de Investigación Sanitaria Gregorio Marañón, Madrid, Spain; 3 Centro de Investigación Biomedica En Red-Enfermedades Respiratorias (CibeRes, CB06/06/0028) and Institut d’Investigacions Biomèdiques August Pi i Sunyer (IDIBAPS), Barcelona, Spain; 4 Center for Biomedical Research CELLEX, School of Medicine, University of Barcelona, Barcelona, Spain; 5 San Gallicano Dermatological Institute IRCCS, 00144 Rome, Italy; 6 Servicio de Microbiología, Hospital Universitario Ramón y Cajal and Instituto Ramón y Cajal de Investigación Sanitaria (IRYCIS), Madrid, Spain; 7 Red Española de Investigación en Patología Infecciosa (REIPI), Madrid, Spain; 8 Group For Biomedical Research in Sepsis (BioSepsis) Hospital Clínico Universitario de Valladolid, Valladolid, Spain; 9 Centro de Investigación Biomedica En Red - Enfermedades Respiratorias (CibeRes, CB06/06/0028), Barcelona, Spain; 10 National Health System, SACYL/IECSCYL, Valladolid, Spain; 11 Servicio de Microbiología y Parasitología Hospital Universitario La Paz, Madrid, Spain; 12 CIBER Enfermedades Respiratorias-CIBERES (CB06/06/0058), Madrid, Spain; 13 Medicine Department, School of Medicine, Universidad Complutense de Madrid, Madrid, Spain

**Keywords:** *Pseudomonas aeruginosa*, Ventilator-associated pneumonia, Virulence genes, Biofilm, *Galleria mellonella*

## Abstract

**Background:**

The objective of this study was to evaluate the virulence of *P. aeruginosa* ventilator-associated pneumonia (VAP) strains (cases) in terms of biofilm production and other phenotypic and genotypic virulence factors compared to *P. aeruginosa* strains isolated from other infections (controls).

**Methods:**

Biofilm production was tested to assess biomass production and metabolic activity using crystal violet binding assay and XTT assay, respectively. Pigment production (pyocyanin and pyoverdine) was evaluated using cetrimide agar. Virulence genes were detected by conventional multiplex PCR and virulence was tested in an in vivo model in *Galleria mellonella* larvae.

**Results:**

We did not find statistically significant differences between VAP and no-VAP strains (*p* > 0.05) regarding biofilm production. VAP strains had no production of pyocyanin after 24 h of incubation (*p* = 0.023). The distribution of virulence genes between both groups were similar (p > 0.05). VAP strains were less virulent than non-VAP strains in an in vivo model of *G. mellonella* (*p* < 0.001).

**Conclusion:**

The virulence of VAP-*Pseudomonas aeruginosa* does not depend on biofilm formation, production of pyoverdine or the presence of some virulence genes compared to *P. aeruginosa* isolated from non-invasive locations. However, VAP strains showed attenuated virulence compared to non-VAP strains in an in vivo model of *G. mellonella*.

**Supplementary Information:**

**Supplementary information** accompanies this paper at 10.1186/s12879-020-05534-1.

## Background

*Pseudomonas aeruginosa* is one of the most common causes of ventilator-associated pneumonia (VAP), with high mortality rates (approximately 13%), prolonged hospital stays and increasing hospital costs [1]. VAP is a nosocomial lung infection that appears 48 h after intubation characterised by new lung infiltrates, signs of systematic infection, changes in the appearance of sputum, leukocytosis and decline in oxygenation [2, 3]. The incidence of VAP ranges between 2 and 16 episodes per ventilator days being around 44% of the episodes caused by *P. aeruginosa* [4].

VAP is caused mainly by multidrug-resistant and extremely drug-resistant strains of *P. aeruginosa*, leading to an increase in treatment failure [5]. Moreover, the activity of antibiotics is restricted by *P. aeruginosa* biofilms that form rapidly on the surface of the endotracheal tube [6]. These microbial communities constitute one of the most important virulence factors of *P. aeruginosa*. However, many more virulence factors are involved in VAP development, such as type III secretory proteins (*exoT*, *exoS*, *exoY*, and *exoU*), which control the expression of exotoxins, quorum-sensing (QS) system proteins (*lasR/lasI* and *rhlR/rhlI*) allowing the communication between cells, elastases (*lasA* and *lasB*) that disrupt tight junctions between host epithelial cells, alginate (*alg* genes), and pigments such as pyoverdine, which up-regulates the transcription of some kinds elastases, and pyocyanin, involved in oxidative stress promoting the alteration of mitochondrial electron transport of the host [1, 7, 8].

Diagnosis and treatment of VAP are still a challenge in clinical settings. Understanding the molecular basis of pathogenicity may lead to new treatment targets and new biomarkers for improving management of VAP. Hence, the aim of this study was to compare the virulence of *P. aeruginosa* strains causing VAP in terms of various factors with that of *P. aeruginosa* strains isolated from other sources of infection.

## Methods

This multicentre study was conducted in the Clinical Microbiology Laboratory of Hospital General Universitario Gregorio Marañón, Madrid, Spain. A total of 90 *P. aeruginosa* strains were collected and they were distributed as follows: 40 different (1 per patient) cases from lower respiratory tract samples of patients with VAP obtained from 5 institutions (4 from Istituti Fisioterapici Ospitalieri, Rome, Italy; 3 from Hospital Ramón y Cajal, Madrid, Spain; 1 from Hospital la Paz, Madrid, Spain; 11 from Hospital Clinic, Barcelona, Spain, and 21 from Hospital Universitario Gregorio Marañón, Spain) and 50 controls from various sites (central venous catheter, urinary tract, blood, faeces, cerebrospinal fluid, joint fluid, cornea, abscesses, skin ulcers, and nephrostomy tubes) obtained from Istituti Fisioterapici Ospitalieri (*n* = 15) and Hospital General Universitario Gregorio Marañón (*n* = 35). Control samples were collected randomly both retrospectively and prospectively. VAP-samples were collected from patients with clinical and microbiological diagnosis of VAP when the last one was confirmed.

All the assays were performed in the Clinical Microbiology Laboratory of Hospital General Universitario Gregorio Marañón, Madrid, Spain. The other centres were responsible of collecting samples and clinical and microbiology data of the samples.

### Biofilm production

Biofilms were assessed as described by our group, with some modifications [9]. Briefly, a loopful of fresh medium with *P. aeruginosa* was inoculated in 20 ml of BHI broth (Brain Heart Infusion) and incubated with shaking at 37 °C for 24 h in an orbital shaker. After 3 cycles of centrifugation-resuspension with PBS (phosphate buffer saline), inoculums were adjusted to 0.5 McFarland (10^8^ cfu/ml) in BHI broth. One hundred microliters were then added to each well of a 96-well plate, and plates were incubated at 37 °C for 24 h. Wells were washed 3 times with PBS, and, once the plates were completely dry, the crystal violet (CV) and the tetrazolium salt XTT [2,3-bis-(2-methoxy-4-nitro-5-sulfophenyl)-2H-tetrazolium-5-carboxanilide] assays were applied.

For biomass quantification, after PBS washes, 200 μl of 99% methanol was used to fix biofilms for 10 min at room temperature. Methanol was discarded, and 125 μl of CV was added for 15 min. The plates were washed with distilled water, and 125 μl of 30% acetic acid was added for 15 min. Absorbance was measured at 550 nm in a spectrophotometer (Biochrom EZ Read 400).

The metabolic activity of the biofilms was evaluated using XTT. Menadione and XTT were mixed immediately before each experiment at a proportion of 1 μl per 10 ml of XTT, and 100 μl was added to each of the wells after PBS wash. Plates were incubated at 37 °C for 2 h in darkness. Absorbance was measured at 492 nm using a spectrophotometer (Biochrom EZ Read 400).

All strains were tested in triplicate using *P. aeruginosa* PA01 (a biofilm-producing strain) as the positive control and sterile BHI broth as the negative control.

Strains were classified as low, moderate, or high biofilm producers using the optical density (OD) as follow: low biofilm producer, OD ≤ 2 × ODc; moderate biofilm producer, 2 × ODc < OD < 4 × ODc; and high biofilm producer, OD ≥ 4 × ODc, where ODc represents 3 standard deviations above the mean of the negative control and OD indicates the mean optical density of the strain [10].

### Pyocyanin and pyoverdine production

**Fig 1:**
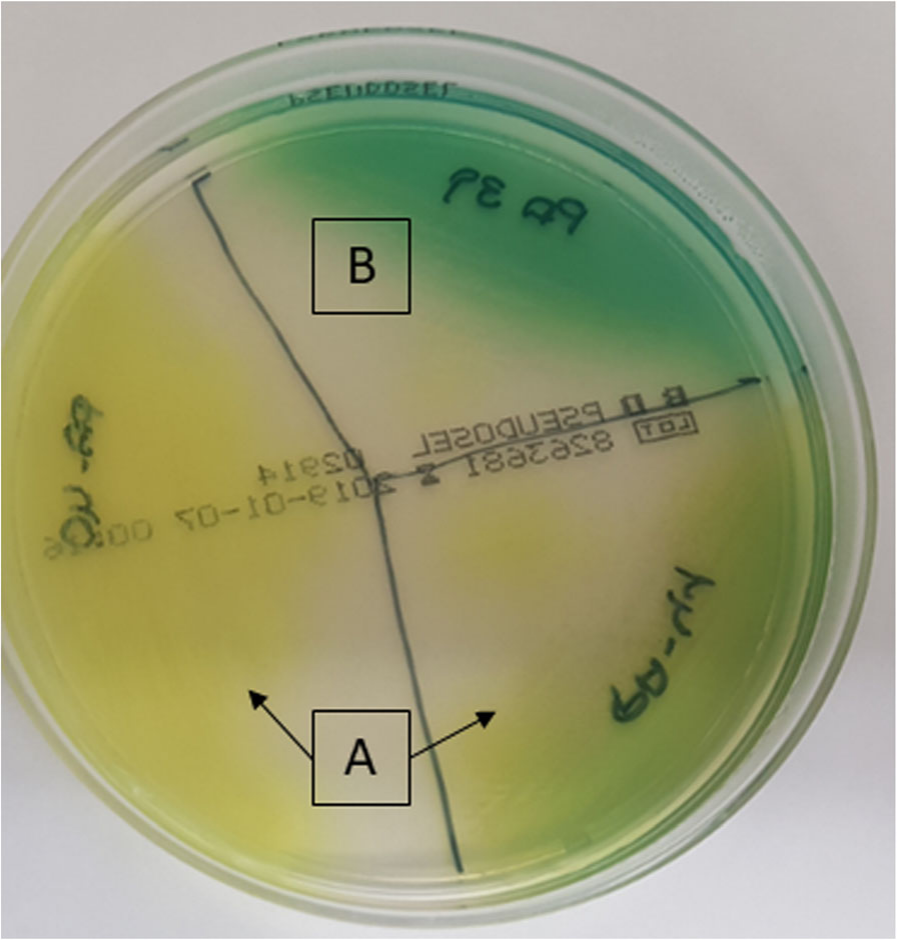
Survival curves for *Galleria mellonella* larvae after infection with *Pseudomonas aeruginosa* strains isolated from patients with VAP (VAP *P. aeruginosa*, *n* = 8) and from patients with other infections (non-VAP *P. aeruginosa*, *n* = 8). PBS was used as a positive control for larvae survival. *Statistically differences were found between *VAP and non-VAP P. aeruginosa* (*p* < 0.001) using the log-rank test

Pigment production was evaluated in cetrimide agar (Pseudosel Agar, BD) by qualitative observation. Inoculated plates were incubated at 37 °C for 24 h. Colonies that appeared fluorescent green, yellow, or brown in colour were considered to be pyoverdine producers. Dark green or blue colonies were considered to be pyocyanin producers (Fig. 1).

### Virulence genes

#### DNA extraction

Strain DNA was extracted using the QIAamp® DNA Mini Kit (250) (QIAGEN) following the manufacturer’s instructions. DNA was eluted in 100 μl of elution buffer and stored at 4 °C.

#### Polymerase chain reaction

We tested the presence or absence of 14 virulence genes—*exoT, exoY, exoS, exoU, algD, algU, plcH, plcN, apr, rhII, rhIR, lasI, lasR*, and *lasB*—by conventional multiplex and uniplex PCR, as described elsewhere [11–13]. Primer sequences [11, 13], amplicon size, and PCR conditions are shown in Table 1. *exoU* primers were designed in this study using the online Primer-Blast program of the National Center for Biotechnology Information. The PCR master mix was composed of nuclease-free water, Phusion U Green Multiplex PCR Master Mix (1X) (Thermo Scientific™), and primers at a final concentration of 0.3 μM. PCR was performed in a thermocycler (GeneAmp PCR system 9700, Applied Biosystems). Strain PA01 was used as a positive control. PA14, which was kindly provided by Dr. Oliver, was used as a positive control for detection of *exoU*.

**Table 1:** Distribution of the presence of virulence genes from *Pseudomonas aeruginosa* strains isolated from lower respiratory tract samples of patients with VAP (cases) and patients with non-VAP infection (controls)

	*algD N (%)*	*algU N (%)*	*plcH N (%)*	*plcN N (%)*	*exoS N (%)*	*exoY N (%)*	*exoT N (%)*	*exoU N (%)*	*apr N (%)*	*lasB N (%)*	*lasI N (%)*	*lasR N (%)*	*rhII N (%)*	*rhRI N (%)*
Controls	50 (100.0)	41 (82.0)	50 (100.0)	50 (100.0)	37 (74.0)	45 (90.0)	50 (100.0)	15 (30.0)	50 (100.0)	50 (100.0)	48 (96.0)	48 (96.0)	38 (76.0)	37 (74.0)
Cases	40 (100.0)	33 (82.5)	40 (100.0)	40 (100.0)	31 (77.5)	39 (97.5)	40 (100.0)	13 (32.5)	39 (97.5)	40 (100.0)	40 (100.0)	39 (97.5)	36 (90.0)	36 (90.0)
Total	90 (100.0)	74 (82.2)	90 (100.0)	90 (100.0)	68 (75.6)	84 (93.3)	90 (100.0)	28 (31.1)	89 (98.9)	90 (100.0)	88 (97.8)	87 (96.7)	74 (82.0)	73 (81.0)

***N*** Number of strains that showed the gene; ***%*** Percentage of strains that possess the gene

No statistically differences in the presence of virulence genes were found between control and case strains (*p* > 0.05, chi-square)

#### Electrophoresis

Fragments were detected by agarose electrophoresis in 2% agarose gels at 170 V for 30 min. *exoS, plcN, lasB*, and *lasR* amplicons were detected using 4% agarose gels at 170 V for 90 min. Ready-to-use GeneRuler 1-kb and 100-bp DNA ladders (Thermo ScientificTM) were applied to size DNA products.

#### Sequencing

Sequencing was performed in PA01 strain to confirm that amplicons corresponded to the studied genes. DNA was purified using the QIAquick® PCR Purification Kit (250) (QIAGEN) following the manufacturer’s instructions. Forward strains were sequenced in the Genomics Department of Hospital General Universitario Gregorio Marañón. Sequences were aligned using the BLAST database to identify the genes.

### In vivo virulence assay

Of the total of 40 cases and 50 controls, we were only able to test 8 randomly chosen strains of each (cases: PA18, 19 21, 29, 35, 38, 52, and 57; controls: PA03 from skin ulcer, PA27, and PA64 from catheters, PA39 from bone biopsy, PA40 from a rectal exudate, PA50 from a nephrostomy, PA56 from cornea, and PA61 from urine) in the *G. mellonella* model owing to difficulties in the supply of larvae.

The *Galleria mellonella* (*G. mellonella*) infection model was applied as described by Marcos-Zambrano et al., with some modifications [14]. Briefly, an inoculum of 103 cfu/ml of *P. aeruginosa* was injected into the hemocoel via the last left proleg of the instar larvae of *G. mellonella* (Bichosa, Salceda de Caselas, Spain) using a Hamilton syringe. Infected larvae were incubated at 37 °C for 24 h. Each experimental group was composed of 10 randomly chosen larvae (330 ± 20 mg) cleaned with 70% ethanol prior to each experiment. Larvae injected and not injected with PBS were used as controls to monitor injection injury. Larvae death was recorded every hour from 16 h after infection to 24 h. Completely melanised larvae and/or completely immobilized larvae were considered dead.

### Statistical analysis

Quantitative variables were expressed as mean (SD), and qualitative variables were reported as percentages. Categorical variables (distribution of high, moderate and low biofilm producer between case and control groups and the presence or absence of virulence genes between groups) were compared using the chi-square test, and continuous variables (absorbance values for CV and XTT assays between control and case groups) were compared using the *t* test. Statistical significance was set at *p* < 0.05. All tests were performed using the statistical program SPSS 21.0 (IBM).

Killing curves were analysed using the Kaplan-Meier method with GraphPad Prism 5.02 software (GraphPad, La Jolla, CA, USA). Differences in survival were compared using the log-rank (Mantel-Cox) test. Statistical significance was set at *p* < 0.05.

### Ethics statement

The strains collected during this study were obtained by regular diagnostic procedures of the involved institutions. This study was approved by the Ethics Committee of the Hospital General Universitario Gregorio Marañón (code: MICRO.HGUGM.2015–075). No informed consent was required.

## Results

### Biofilm production

Of the 90 strains studied, 76 (84.4%), 13 (14.5%), and 1 (1.1%) strains were high, moderate, and low biomass producers, respectively. As for the metabolic activity of biofilms, the distribution of high, moderate, and low producers was, 17 (18.9%), 34 (37.8%), and 39 (43.3%), respectively. Distribution of strains by biofilm production classified by cases and controls is reported in Table 2. No statistically significant differences in biofilm production were found with respect to biomass production or metabolic activity between cases and controls (*p* > 0.05).

**Table 2:** Primer sequences, fragment size, and PCR conditions for detection of virulence genes in *Pseudomonas aeruginosa*

Gene	Primer sequence (5′➔3′)	Fragment size (bp)	Annealing T (°C)
*algD*	F: AAGGCGGAAATGCCATCTCC	290	60
	R: AGGGAAGTTCCGGGCGTTTG		
*exoY*	F: TATCGACGGTCATCGTCAGGT	1035	
	R: TTGATGCACTCGACCAGCAAG		
*plcH*	F: GCACGTGGTCATCCTGATGC	608	
	R: TCCGTAGGCGTCGACGTAC		
*rhIR*	F: TGCATTTTATCGATCAGGGC	133	57
	R: CACTTCCTTTTCCAGGACG		
*lasI*	F: CGTGCTCAAGTGTTCAAGG	295	
	R: TACAGTCGGAAAAGCCCAG		
*apr*	F: TGTCCAGCAATTCTCTTGC	1017	
	R: CGTTTTCCACGGTGACC		
*exoT*	F: CAATCATCTCAGCAGAACCC	1159	
	R: TGTCGTAGAGGATCTCCTG		
*rhII*	F: TTCATCCTCCTTTAGTCTTCCC	155	
	R: TTCCAGCGATTCAGAGAGC		
*exoS*	F: CGTCGTGTTCAAGCAGATGGTGCTG	444	65
	R: CCGAACCGCTTCACCAGGC		
*plcN*	F: TCCGTTATCGCAACCAGCCCTACG	481	
	R: TCGCTGTCGAGCAGGTCGAAC		
*lasB*	F: GGAATGAACGAAGCGTTCTCCGAC	284	
	R: TGGCGTCGACGAACACCTCG		
*lasR*	F: AAGTGGAAAATTGGAGTGGAG	130	
	R: GTAGTTGCCGACGACGATGAAG		
*algU*	F: CGCGAACCGCACCATCGCTC	1500	66
	R: GCCGCACGTCACGAGC		
*exoU*	F: TACCAGGTACGGCCATGTTC	575	59
	R: ACGCTCTGAAGCCTGAAGAC		

***Bp*** Base pairs; ***F*** Forward; ***R*** Reverse; ***T*** Temperature

PCR was performed following the manufacturer’s instructions

### Pyocyanin and pyoverdine production

Pigment production is another virulence factor of *P. aeruginosa*. In our collection, 74.4% of the strains secreted pyoverdine, 7.8% pyocyanin, 10% both pigments, and 7.8% neither. Pyoverdine was produced more frequently than pyocyanin in VAP strains (92.5% vs 7.5%, respectively) (*p* < 0.001). Production of pyocyanin was more often found in non-VAP isolates (26.0%) than in VAP isolates (7.5%) (*p* = 0.023). All the strains that produced both pigments or neither pigment belonged to the control group.

### Virulence genes

All the strains presented at least 10 virulence genes. *algD, lasB, plcN, plcH*, and *exoT* were present in all the strains. *Apr, lasI, lasR*, and *exoY* were found in 99, 98, 97, and 93% of the strains, respectively. A lower incidence was obtained for *rhII* (82%), *rhRI* (81%), *algU* (82%), and *exoS* (76%). *exoU* was present in only 28 (31.1%) strains. One strain presented *lasI* but not *lasR*. The same phenomenon was observed in another strain, in which *rhII* was present, although its regulatory gene (*rhIR*) was absent. The distribution of virulence genes did not differ significantly between cases and controls (*p* > 0.05) (Table 3). However, a tendency towards absence of *rhlI* and *rhlR* was observed in control strains (*p* = 0.084 and *p* = 0.054, respectively).

**Table 3:** Distribution and classification of the whole collection of *Pseudomonas aeruginosa* strains according to their capacity to form biofilms in terms of biomass production and metabolic activity

	CV			XTT		
	Low producers	Moderate producers	High producers	Low producers	Moderate producers	High producers
**Controls, N (%)**	0 (0.0)	8 (8.9)	42 (46.7)	18 (20.0)	20 (22.2)	12 (13.3)
**Cases, N (%)**	1 (1.1)	5 (5.6)	34 (37.7)	21 (23.0)	14 (15.6)	5 (5.6)
**Total, N (%)**	1 (1.1)	13 (14.5)	76 (84.4)	39 (43.3)	34 (37.8)	17 (18.9)

*N* Number of strains; *%* Percentage of strains; *CV* Crystal violet; *XTT* Tetrazolium salt

### In vivo virulence assay

**Fig 2:**
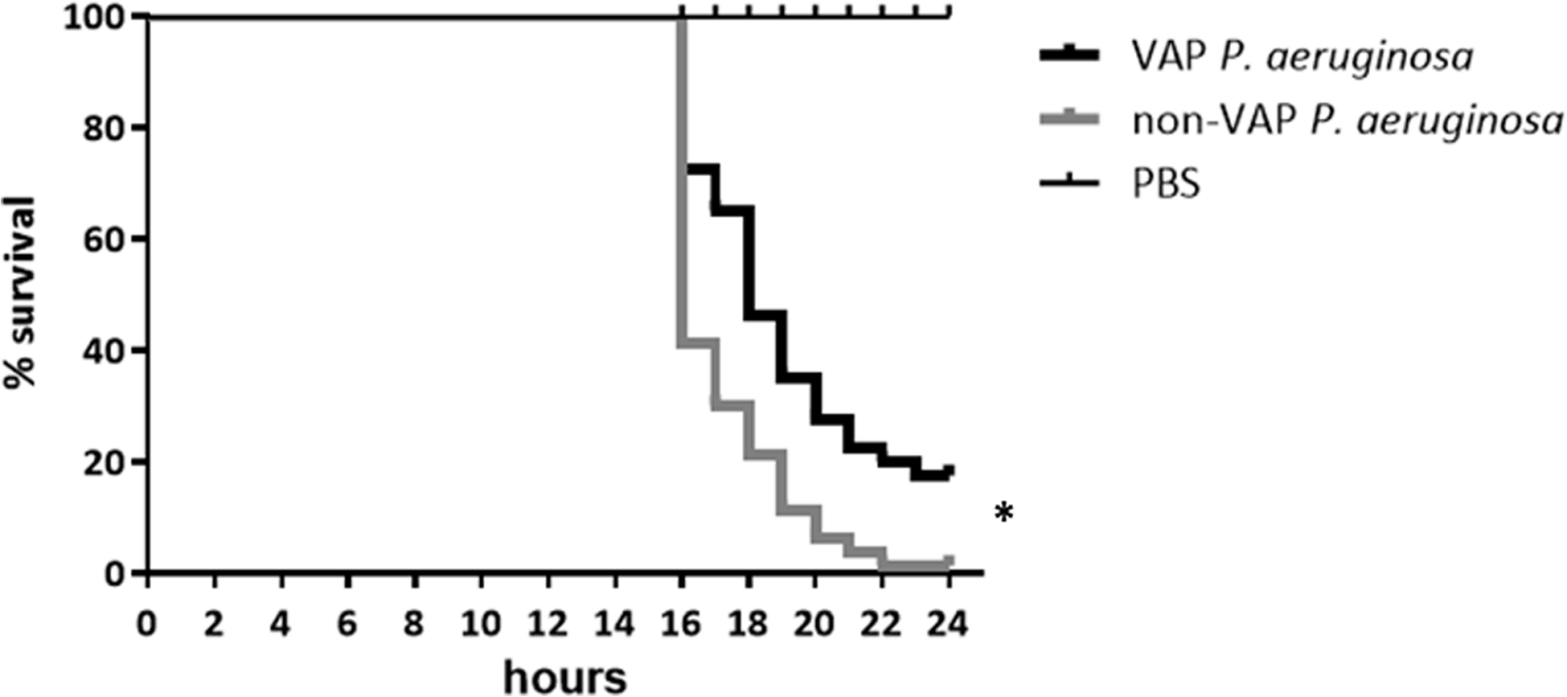
Classification of pyoverdine and pyocyanin producer by qualitative observational method. **a**, pyoverdine producer strain; **b**, pyocyanin producer strain

Although only 8 strains from each group were compared in terms of virulence and no correlation between genetic background and the phenotype in vivo was observed (data not shown), we found significant differences between larvae mortality (*p* < 0.001). The median (IQR) time of survival for cases was 18 h (3.0) and for control strains 16 h (2.0) (Fig. 2). This means that *P. aeruginosa* strains causing VAP were less virulent than those isolated from other origins.

## Discussion

Our results show that the underlying pathogenicity mechanism of *P. aeruginosa* VAP is not restricted to biofilm production and specific virulence genes. However, VAP isolates did not produce pyocyanin after 24 h of incubation and showed lower virulence in the *G. mellonella* model than non-VAP strains.

Many studies have focused on the management of VAP owing to its impact in clinical settings [15–17]. However, few studies investigate the pathogenesis of *P. aeruginosa* VAP [7, 18, 19]. Hence, this study is the first to characterise a large sample of *P. aeruginosa* strains isolated from patients with VAP in comparison with strains from other sources from various hospitals in Europe, thus contributing to our understanding of the role of virulence mechanisms of *P. aeruginosa* in the pathogenesis of VAP.

*P. aeruginosa* is characterised by its capacity to form robust biofilms that play a substantial role in many human infections such as cystic fibrosis (CF), chronic wound infection, chronic otitis media, and implanted medical device infection [20]. One of the main risk factors for VAP is the presence of an endotracheal tube into which *P. aeruginosa* forms biofilms and enters the lower respiratory tract to cause infection [6]. We showed that in terms of biomass, high-biofilm-producing strains are implicated not only in VAP, but also in many other kinds of infections, although with no statistically significant differences, as reported by Rybtke et al. [20]. Moreover, the finding that most of the strains showed moderate or low metabolic activity when grown in biofilms may be considered a characteristic of *P. aeruginosa* biofilms. This characteristic confers intrinsic antibiotic resistance to biofilms, especially for antibiotics that target cellular processes associated with metabolic activity and active growth, thus rendering *P. aeruginosa* infections difficult to treat [20, 21].

Production of pigments such as pyoverdine and pyocyanin also play a key role in the virulence of *P. aeruginosa* [7]. Strains isolated from VAP patients showed a correlation with the absence of production of pyocyanin, and all the strains secreted pyoverdine after 24 h of incubation. Pyoverdine is a siderophore that chelates the iron needed for survival of *P. aeruginosa* [7]. The role of pyoverdine could explain why it is secreted by all VAP strains, as it is necessary for biofilm formation and hence for colonization of the endotracheal tube. However, pyocyanin is a redox compound involved in damage to lung epithelium and has been widely studied in cystic fibrosis [22, 23]. Although pyocyanin was not produced in VAP strains in our study, we hypothesized that pigment production could be a 2-step process, as pyocyanin has been reported to play an essential role in the pathogenesis of pneumonia [23]. Pyoverdine is necessary for colonization and biofilm formation and could be secreted initially, before synthetization of pyocyanin, leading to lung damage and pneumonia.

As for virulence factors, biofilm formation is regulated by many genes, such as *algR* (*algU* and *algD*), *psl,* and *pel*, which play a role in mucoidity and robustness of the extracellular matrix [21]. After detecting *algU* and *algD*, both of which are involved in alginate production, we observed that *algD* was present in all the strains, whereas the presence of *algU* was lower. *algU* is an extracytoplasmic sigma factor that is normally sequestered in the cytoplasmic membrane. Under specific conditions, it is released and activates overproduction of alginate [24]. This virulence factor plays a key role in the pathogenesis of cystic fibrosis by contributing to the mucoid phenotype of strains [24].

In addition to biofilm formation, the principal virulence factors of *P. aeruginosa* are elastase, phospholipase C, protease A, exotoxins and cytotoxins, flagella and pili, pigment production, and QS regulatory system proteins, which regulate both virulence factor transcription and biofilm formation [25]. Although it is well established that lung damage is caused by these proteins during pneumonia, their importance as virulence factors in VAP is unknown [8, 26]. We observed that there were no statistically significant differences in the presence of virulence genes between case and control strains. Our results corroborate the conclusion of Wolfgang et al., who maintained that the *P. aeruginosa* genome is highly conserved, at least for the 14 genes of this study, independently of the source of isolation [27].

Finally, we investigated the virulence of *P. aeruginosa* in an in vivo model based on *G. mellonella* larvae, which have been used widely to analyse the virulence of many microorganisms owing to their specific advantages [28]. We observed that VAP strains were less virulent than strains isolated from other infections, as previously reported by Wang et al. in an outbreak of VAP in a hospital in China, even though all strains presented similar patterns of virulence genes [29], indicating that virulence may be controlled by transcriptional regulation. Our results suggest that depending on the site of infection, particularly in the lung in VAP, modification of some virulence genes of *P. aeruginosa* combined with mutation of QS genes, as reported by Köhler et al., may play a key role in adaptation by bacteria [30]. Furthermore, although lung immune response is still under research, it plays a key role in the infection of *P. aeruginosa* in VAP patients [31]. Hence, alternative approaches to the diagnosis and treatment of VAP may rely on the development of an exhaustive epigenetic profile of VAP strains and a deep study of the lung immune system in mechanical ventilator.

Although ours is the first study to provide important information on the distribution of virulence factors in a wide range of VAP isolates of *P. aeruginosa* collected from hospitals in Spain and Italy, it is subject to limitations such as the functionality of the genes was not investigated by sequencing. However, the most important was the low number of strains tested in the in vivo model. However, although we only tested 8 strains in each group, we were able to find statistically significant differences. Increasing the sample size would only have increased the statistical power.

## Conclusion

We demonstrated that the virulence and pathogenesis of *P. aeruginosa* in VAP infection does not depend on the presence of virulence genes or biofilm formation. However, these isolates were less virulent in vivo. Future studies are needed to elucidate how virulence is regulated during VAP and hence, improve the diagnosis and treatment of the disease.

## Supplementary Information

**Additional file 1.** Supplementary material

## Data Availability

The datasets used and/or analysed during the current study are available from the corresponding author on reasonable request.

## Abbreviation

VAP: Ventilator-associated pneumonia

## References

[1] Aykac K, Ozsurekci Y, Tanir BS (2017). Future directions and molecular basis of ventilator associated pneumonia. Can Respir J.

[2] Kalanuria AA, Ziai W, Mirski M (2014). Ventilator-associated pneumonia in the ICU. Crit Care.

[3] Kalil AC, Metersky ML, Klompas M, Muscedere J, Sweeney DA, Palmer LB (2016). Management of Adults with Hospital-acquired and Ventilator-associated Pneumonia: 2016 clinical practice guidelines by the Infectious Diseases Society of America and the American Thoracic Society. Clin Infect Dis.

[4] Sarda C, Fazal F, Rello J (2019). Management of ventilator-associated pneumonia (VAP) caused by resistant gram-negative bacteria: which is the best strategy to treat?. Expert Rev Respir Med.

[5] Maurice NM, Bedi B, Sadikot RT (2018). Pseudomonas aeruginosa biofilms: host response and clinical implications in lung infections. Am J Respir Cell Mol Biol.

[6] Fernandez-Barat L, Torres A. Biofilms in ventilator-associated pneumonia. Future Microbiol. 2016;11:1599–610. 10.2217/fmb-2016-0040 27831764

[7] Berra L, Schmidt U, Wiener-Kronish J (2010). Relationship between virulence factors and outcome of ventilator-associated pneumonia related to Pseudomonas aeruginosa. Curr Respir Med Rev.

[8] Gellatly SL, Hancock RE (2013). Pseudomonas aeruginosa: new insights into pathogenesis and host defenses. Pathog Disease.

[9] Alonso B, Lucio J, Perez-Granda MJ, Cruces R, Sanchez-Carrillo C, Bouza E, et al. Does biomass production correlate with metabolic activity in *Staphylococcus aureus*? J Microbiol Methods. 2016;131:110–12. 10.1016/j.mimet.2016.10.011 27776997

[10] Stepanovic S, Vukovic D, Hola V, Di Bonaventura G, Djukic S, Cirkovic I (2007). Quantification of biofilm in microtiter plates: overview of testing conditions and practical recommendations for assessment of biofilm production by staphylococci. APMIS.

[11] Fazeli N, Momtaz H (2014). Virulence gene profiles of multidrug-resistant Pseudomonas aeruginosa isolated from Iranian hospital infections. Iran Red Crescent Med J.

[12] Gholami A, Majidpour A, Talebi-Taher M, Boustanshenas M, Adabi M (2016). PCR-based assay for the rapid and precise distinction of Pseudomonas aeruginosa from other Pseudomonas species recovered from burns patients. J Prev Med Hyg.

[13] Sabharwal N, Dhall S, Chhibber S, Harjai K (2014). Molecular detection of virulence genes as markers in Pseudomonas aeruginosa isolated from urinary tract infections. Int J Mol Epidemiol Genet.

[14] Marcos-Zambrano LJ, Puig-Asensio M, Perez-Garcia F, Escribano P, Sanchez-Carrillo C, Zaragoza O, et al. *Candida guilliermondii* complex is characterized by high antifungal resistance but low mortality in 22 cases of candidemia. Antimicrob Agents Chemother. 2017;61(7):e00099–17. 10.1128/AAC.00099-17 PMC5487632 28438935

[15] Weiss E, Essaied W, Adrie C, Zahar JR, Timsit JF (2017). Treatment of severe hospital-acquired and ventilator-associated pneumonia: a systematic review of inclusion and judgment criteria used in randomized controlled trials. Crit Care.

[16] Landelle C, Nocquet Boyer V, Abbas M, Genevois E, Abidi N, Naimo S (2018). Impact of a multifaceted prevention program on ventilator-associated pneumonia including selective oropharyngeal decontamination. Intensive Care Med.

[17] Tokmaji G, Vermeulen H, Muller MC, Kwakman PH, Schultz MJ, Zaat SA. Silver-coated endotracheal tubes for prevention of ventilator-associated pneumonia in critically ill patients. Cochrane Database Syst Rev. 2015(8):Cd009201. 10.1002/14651858.CD009201.pub2 PMC6517140 26266942

[18] Sadikot RT, Blackwell TS, Christman JW, Prince AS (2005). Pathogen-host interactions in Pseudomonas aeruginosa pneumonia. Am J Respir Crit Care Med.

[19] Kohler T, Guanella R, Carlet J, van Delden C (2010). Quorum sensing-dependent virulence during Pseudomonas aeruginosa colonisation and pneumonia in mechanically ventilated patients. Thorax..

[20] Rybtke M, Hultqvist LD, Givskov M, Tolker-Nielsen T (2015). Pseudomonas aeruginosa biofilm infections: community structure, antimicrobial tolerance and immune response. J Mol Biol.

[21] Lee K, Yoon SS (2017). Pseudomonas aeruginosa biofilm, a programmed bacterial life for fitness. J Microbiol Biotechnol.

[22] Rada B, Leto TL (2013). Pyocyanin effects on respiratory epithelium: relevance in Pseudomonas aeruginosa airway infections. Trends Microbiol.

[23] Lau GW, Hassett DJ, Ran H, Kong F (2004). The role of pyocyanin in Pseudomonas aeruginosa infection. Trends Mol Med.

[24] Stacey SD, Pritchett CL (2016). Pseudomonas aeruginosa AlgU contributes to posttranscriptional activity by increasing rsmA expression in a mucA22 strain. J Bacteriol.

[25] Azam MW, Khan AU. Updates on the pathogenicity status of *Pseudomonas aeruginosa*. Drug Discov Today. 2019;24(1):350–59. 10.1016/j.drudis.2018.07.003 30036575

[26] Sawa T (2014). The molecular mechanism of acute lung injury caused by Pseudomonas aeruginosa: from bacterial pathogenesis to host response. J Intensive Care.

[27] Wolfgang MC, Kulasekara BR, Liang X, Boyd D, Wu K, Yang Q (2003). Conservation of genome content and virulence determinants among clinical and environmental isolates of Pseudomonas aeruginosa. Proc Natl Acad Sci U S A.

[28] Tsai CJ, Loh JM, Proft T (2016). Galleria mellonella infection models for the study of bacterial diseases and for antimicrobial drug testing. Virulence.

[29] Wang K, Chen YQ, Salido MM, Kohli GS, Kong JL, Liang HJ, et al. The rapid in vivo evolution of *Pseudomonas aeruginosa* in ventilator-associated pneumonia patients leads to attenuated virulence. Open Biol. 2017;7(9):170029. 10.1098/rsob.170029 PMC5627047 28878043

[30] Kohler T, Buckling A, van Delden C (2009). Cooperation and virulence of clinical Pseudomonas aeruginosa populations. Proc Natl Acad Sci U S A.

[31] De Winter FHR, s Jongers B Bielen K, Mancuso D, Timbermont L, Lammens C, et al. Mechanical ventilation impairs IL-17 cytokine family expression in ventilator-associated pneumonia. 2019;20(20):5072. 10.3390/ijms20205072 PMC6829394 31614857

