# Correction to: Characterization of the virulence of *Pseudomonas aeruginosa* strains causing ventilator-associated pneumonia

**DOI:** 10.1186/s12879-020-05691-3

**Published:** 2020-12-11

**Authors:** Beatriz Alonso, Laia Fernández-Barat, Enea Gino Di Domenico, Mercedes Marín, Emilia Cercenado, Irene Merino, Manuela de Pablos, Patricia Muñoz, María Guembe

**Affiliations:** 1 Department of Clinical Microbiology and Infectious Diseases, Hospital General Universitario Gregorio Marañón, Madrid, Spain; 2 Instituto de Investigación Sanitaria Gregorio Marañón, Madrid, Spain; 3 Centro de Investigación Biomedica En Red-Enfermedades Respiratorias (CibeRes, CB06/ 06/0028) and Institut d’Investigacions Biomèdiques August Pi i Sunyer (IDIBAPS), Barcelona, Spain; 4 Center for Biomedical Research CELLEX, School of Medicine, University of Barcelona, Barcelona, Spain; 5 San Gallicano Dermatological Institute IRCCS, 00144 Rome, Italy; 6 Servicio de Microbiología, Hospital Universitario Ramón y Cajal and Instituto Ramón y Cajal de Investigación Sanitaria (IRYCIS), Madrid, Spain; 7 Red Española de Investigación en Patología Infecciosa (REIPI), Madrid, Spain; 8 Group For Biomedical Research in Sepsis (BioSepsis) Hospital Clínico Universitario de Valladolid, Valladolid, Spain; 9 Centro de Investigación Biomedica En Red - Enfermedades Respiratorias (CibeRes, CB06/06/0028), Barcelona, Spain; 10 National Health System, SACYL/IECSCYL, Valladolid, Spain; 11 Servicio de Microbiología y Parasitología Hospital Universitario La Paz, Madrid, Spain; 12 CIBER Enfermedades Respiratorias-CIBERES (CB06/06/0058), Madrid, Spain; 13 Medicine Department, School of Medicine, Universidad Complutense de Madrid, Madrid, Spain

**Correction to: BMC Infect Dis (2020) 20:909**

**https://doi.org/10.1186/s12879-020-05534-1**

Following publication of the original article [1], the authors identified an error in the caption of Figs. 1 and 2: these should be inverted.

The correct captions are given below.

The correct caption for Fig. 1 is: "Classification of pyoverdine and pyocyanin producer by qualitative observational method. a, pyoverdine producer strain; b, pyocyanin producer strain"

The correct caption for Fig. 2 is: "Survival curves for Galleria mellonella larvae after infection with *Pseudomonas aeruginosa* strains isolated from patients with VAP (VAP *P. aeruginosa*, *n* = 8) and from patients with other infections (non-VAP *P. aeruginosa*, *n* = 8). PBS was used as a positive control for larvae survival. *Statistically differences were found between VAP and non-VAP *P. aeruginosa* (*p* < 0.001) using the log-rank test”.

The original article [1] has been updated.
